# Q Fever Presented as a Large Retroperitoneal Pseudotumoral Mass

**DOI:** 10.1155/2017/4076159

**Published:** 2017-11-15

**Authors:** Behdokht Nowroozizadeh, Negar Haghighi Mehmandari, Nicolas Gallegos, Mari Perez-Rosendahl, Di Lu

**Affiliations:** ^1^Departments of Pathology, UC Irvine Medical Center, UC Irvine School of Medicine, Orange, CA, USA; ^2^Departments of Pathology, UCLA Medical Center, 10833 Le Conte Ave., Los Angeles, CA 90095, USA

## Abstract

**Background:**

Q fever is an infection caused by* Coxiella burnetii*, an intracellular organism. Acute infection is most often a benign and asymptomatic process; however, some individuals may go on to develop subacute and persistent localized symptomatic Q fever. As such, the clinical and histopathologic findings of Q fever are widely variable and may be missed if clinical suspicion is not high.

**Case Presentation:**

Herein we report the first case of* C. burnetii* infection presenting as an isolated retroperitoneal mass. A 61-year-old male underwent axillary-bifemoral bypass surgery. His postoperative course was complicated by the discovery of a large retroperitoneal mass.

**Conclusion:**

Clinical and histopathologic findings of* Coxiella burnetii* infection are variable and can be deceiving. These are often nonspecific, especially in its persistent localized infectious stages.

## 1. Introduction

Q fever is a zoonotic infection caused by the obligate intracellular gram-negative pathogen* Coxiella burnetii*. Acute or persistent localized infection can occur, often with variable clinical and pathological features [1].

Acute Q fever infection is asymptomatic in 60% of cases but can manifest with nonspecific symptoms like fever, myalgia, malaise, hepatitis, and pneumonia [2]. Common manifestations of persistent localized Q fever infection include endocarditis, infectious thoracoabdominal aortic aneurysm, vascular graft infection, bone lesions, pulmonary infection, and granulomatous hepatitis. Encephalitis, pericarditis, and myocarditis have also been reported [3, 4]. In addition, there are rare reports of* Coxiella burnetii* infection presenting as mass lesions in the lung, mimicking malignant tumors radiographically [5, 6].

In our case, the patient presented with a large retroperitoneal mass that was clinically and radiographically suspicious for a sarcoma. CT-guided biopsy of the mass showed extensive ischemic-like necrosis involving skeletal muscle and fibroconnective tissue. Typical histologic characteristics of infection or inflammation were absent. Clinical work-up with serologic studies, however, revealed a high titer of* C. burnetii* antibody.

## 2. Case Presentation

A 61-year-old Vietnamese male with a past medical history of coronary artery disease, peripheral vascular disease, and abdominal aortic aneurysm underwent an axillary-bifemoral bypass and bilateral lower extremity thromboembolectomy. He was referred to our tertiary care center when his preoperative course was complicated by malaise, productive cough, and weight loss of 5 pounds over 5 weeks. The patient was a smoker and was currently employed as an electronics engineer. He denied night sweats, tuberculosis exposure, foreign travel, or animal contact in the past six months. He did report a cattle field along his route to work.

**Figure 1:**
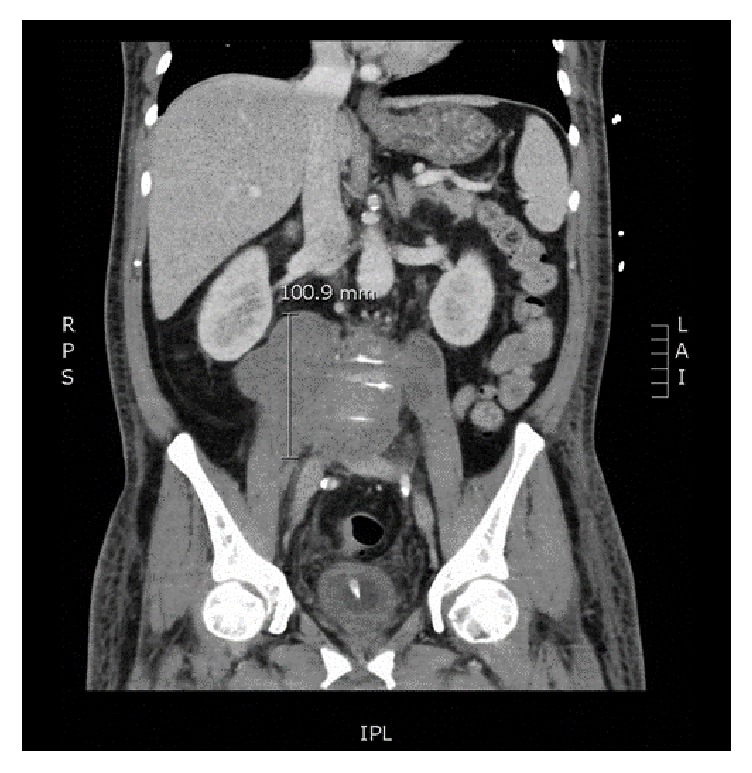
Abdominal CTA showing a lobulated low-density soft tissue mass within the retroperitoneum with involvement of the bilateral psoas muscles.

Abnormal laboratory values were as follows: erythrocyte sediment rate more than 120 mm/hr, white blood cell count of 18.8 × 10^9^ per liter (L), 82% neutrophils, 12% lymphocytes, 6% monocytes, and hemoglobin of 8.5 g/dL. Computed tomography (CT) scan of the chest showed bilateral ground glass opacities, most prominent in the right lower lobe, consistent with pulmonary edema. There was no evidence of a mass lesion in the chest. Abdominal computed tomography angiography (CTA) showed a retroperitoneal 13.1 × 10 × 5 cm hypodense mass lesion at the L3-L5 level, extending to the bilateral psoas muscles (Figure 1). The mass was associated with bone destruction and sclerosis of the adjacent L4 vertebral body.

**Figure 2:**
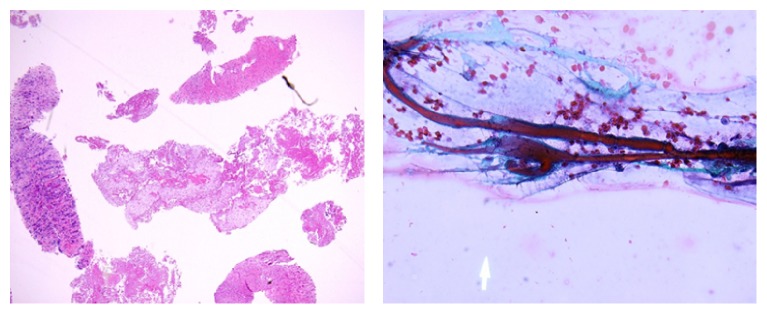
Fine needle aspiration revealed benign skeletal muscle, fibrous tissue, and few fragments of necrotic material.

Magnetic resonance imaging of the abdomen confirmed a 13.9 × 5.1 × 10.3 cm lobulated retroperitoneal mass extending from the left to right psoas muscle. Possible invasion to the posterior wall of the infrarenal abdominal aorta and ventral L4 vertebral body was seen, raising concern for an infectious or neoplastic process. QuantiFERON-TB testing was negative, as was serologic testing for* Treponema pallidum*,* Cryptococcus*,* Histoplasma*,* Coccidioides*, and* Brucella*. Semiquantitative indirect immunofluorescent assay showed high anti-*C. burnetii* antibody titers: phase I IgG titer of 1 : 16384 with reference range of <1 : 16 (performed by ARUP laboratories) and phase II IgG titer of 1 : 4096 with reference range of <1 : 16 (performed by ARUP laboratories). CT-guided core biopsy was performed to evaluate the retroperitoneal mass, which revealed ischemic necrosis involving skeletal muscle and fibro connective tissue (Figure 2).

Acid-fast bacilli (AFB) and Gomori methenamine silver (GMS) stains were negative for acid-fast bacilli and fungal organisms. Immunohistochemistry for CD34, CD117, S100, and pankeratin was negative. Desmin and myogenin immunostains were positive in benign skeletal muscle.

Because there was a high index of suspicion for a malignant process, a second CT-guided core biopsy was performed one week later; histopathological findings were reminiscent of the first biopsy and showed no evidence of malignancy. At this point, the overall clinical and histopathological findings raised a high index of suspicion for* C. burnetii *infection. Tissue from the core biopsy was sent to the Health Department of Orange County, California, where a direct PCR test for* C. burnetii *confirmed the diagnosis. The patient began treatment for Q fever with doxycycline 100 mg twice a day and hydroxychloroquine 600 mg daily. He showed marked clinical improvement with this treatment regime. Three months later, a repeat CT scan showed significant decrease in the size of the retroperitoneal mass, with maximum dimension decrease to 6.5 cm, down from 13.9 cm at presentation. The patient recently completed an 18 month treatment course without complication. Final posttreatment imaging and serology are not available at our institution for review.

## 3. Discussion

Various case reports exist with respect to atypical presentations of Q fever. Q fever has reportedly been associated with abdominal aortic aneurysm, aortoenteric fistula, lower limb fistula, vascular graft infection, vertebral body erosions, CNS infection, fever of unknown origin, and possible pancreatitis [3, 4, 7–10]. Mass lesions are one of the rarest presentations of Q fever. To our knowledge, there have only been 2 previously reported cases, both lung masses [5, 6]. Janigan and Marrie reported a case, which presented with a single lung mass near the left para-aortic region, detected by chest X-ray and CT scan. A left upper lobectomy was performed. Histopathologic examination revealed small edematous bronchioles occluded by fibroblasts and sparse collagen intermixed with inflammatory cells. The diagnosis of Q fever was rendered after electron microscopy revealed the organism [5]. Lipton et al. presented a case of a right lower lobe lung mass with irregular borders seen on imaging. Bronchoscopy and fine needle aspiration failed to provide a definite diagnosis; the diagnosis of* C. burnetii *was made based on serologic markers [6].

Typically, the inflammatory/immune response to Q fever results in granulomatous lesions, most commonly seen in the lungs, liver, and bone marrow. The characteristic finding of Q fever is the so-called donut granuloma or fibrin ring granuloma, which consists of dense fibrin rings surrounding central lipid vacuoles. In persistent localized infection, pathologic findings may also include lymphocytic infiltration and foci of spotty necrosis [2].

To our knowledge, this is the first case reported of Q fever presenting as a solitary retroperitoneal mass. Due to the clinical suspicion for malignancy, CT-guided biopsy was performed twice. Histopathologic findings for both procedures revealed ischemic-like tissue necrosis without significant inflammation or granulomatous change. This is not typical of any infectious process but may be a unique finding for Q fever. Although the patient has no apparent direct exposure to animals, the cattle field located on his way to work could be regarded as an indirect exposure. In our case, the suspicion for Q fever was raised by the elevated titer, and the diagnosis was confirmed by PCR performed on the biopsy tissue. The clinical response to antibiotic therapy further confirmed the infectious nature of the mass.

## 4. Conclusion

While rare cases of Q fever presenting as inflammatory pseudotumor in the lung have been reported, we report the first case of Q fever presenting as a pseudotumor in the retroperitoneum and describe the major diagnostic challenges. Collectively, the histopathological findings for these pseudotumors associated with* C. burnetii *have not been specific, highlighting the need for heightened awareness of this rare presentation. Our case showed ischemic-like necrosis without inflammation or granulomas, which may be a unique finding. Recognition of this finding in correlation with serologic and molecular studies may help to provide accurate diagnosis in challenging cases of* C. burnetii *infection.
